# The utility of personal activity trackers (Fitbit Charge 2) on exercise capacity in patients post acute coronary syndrome [UP-STEP ACS Trial]: a randomised controlled trial protocol

**DOI:** 10.1186/s12872-017-0726-8

**Published:** 2017-12-29

**Authors:** Jason Nogic, Paul Min Thein, James Cameron, Sam Mirzaee, Abdul Ihdayhid, Arthur Nasis

**Affiliations:** MonashHeart, Monash Health and Monash Cardiovascular Research Centre, 246 Clayton Road, Clayton, Melbourne, VIC 3168 Australia

**Keywords:** Fitbit, Personal activity tracker, Cardiac rehabilitation, Acute coronary syndrome

## Abstract

**Background:**

The benefits of physical activity and cardiovascular rehabilitation on the reduction of cardiovascular risk are well documented. Despite this, significant barriers and challenges remain in optimizing patient risk factors post acute coronary syndromes (ACS) and ensuring patient compliance. Consumer wearable personal activity trackers represent a cost effective and readily available technology that may aid in this endeavour.

**Methods:**

UP-STEP ACS is a prospective single-blinded, two-arm, parallel, randomized control trial with an aim to enrol 200 patients all undertaking cardiac rehabilitation. It will assess the affect that personal activity monitors have on change in exercise capacity in patients post acute coronary syndromes primarily measured by a six-minute walk test (6MWT). Secondary end points will be the improvement in other cardiovascular risk factors, namely; blood lipid and glucose levels, weight, waist circumference, along with mood, quality of life and cardiac rehabilitation adherence. Patients will be randomized to either receive a personal activity tracker or standard post hospital care during their index event. After the 8- week intervention period, patients will return for a clinical review and repeat of baseline assessments including the 6MWT.

**Discussion:**

The utility and impact on exercise capacity of personal activity trackers in patient’s post-acute coronary syndrome has not been assessed. This study aims to add to the scientific evidence emerging regarding the clinical utility and validity of these devices in different patient population groups. If proven to be of benefit, these devices represent a cost effective, easily accessible technology that could aid in the reduction of cardiovascular events.

**Trial registration:**

The trial has been registered with the Australian New Zealand Clinical Trials Registry (ANZCTR). The registration number is ACTRN12617000312347 (28/02/2017).

## Background

The increased understanding of cardiovascular risk factors and the importance of aggressive primary and secondary prevention measures have aided significantly in the reduction of cardiovascular disease and events. However, despite the evidence, the application of these interventions is far from optimal [1–3]. Coronary heart disease (CHD) still represents the leading cause of mortality and morbidity in Australia with almost 20,000 deaths in 2013 attributed to CHD [4]. This is also reflected globally with the World Health Organisation (WHO) attributing 8.76 million deaths, or 15.5% of the estimated 56.4 million deaths in 2015 to CHD [5].

Numerous observational studies have shown that there is an increased risk of recurrent cardiac events in patients who are known to have CHD and in particular those who have survived an acute coronary syndromes (ACS), with new events occurring most commonly within the first year following the index event [6]. Australian data has estimated that 34% of all hospital admissions for ACS are repeat events with this representing a significant burden on the health care system economically, and a poor prognostic marker for patients individually [7].

Aggressive secondary prevention is vital in reducing the risk of recurrent events and forms the pillar of secondary risk reduction. From a pharmacological perspective, guidelines are readily accessible and routinely followed immediately post cardiac event, however there remains inherent challenges with compliance and follow-up. The recent TRANSLATE-ACS study [8] showed that 31% of myocardial infarction (MI) patients had stopped taking at least 1 medication by 6 months, with other studies showing dosage is often suboptimal post discharge with only 1 in 3 patients within the suggested dosage target and only 25% of patients prescribed a dosage increase after discharge [9]. Therefore, close patient follow-up, education and appropriate dose titration is vital.

Non-pharmacological secondary prevention is generally targeted at lifestyle modification, with the most common interventions focused around weight loss, smoking cessation, cardiovascular education programs, implementation of a healthy diet and regular physical activity and psychosocial support. Cardiac rehabilitation (CR), a comprehensive evidence based vehicle used to deliver much of the education, engagement and monitoring of lifestyle modifications have been shown to have a significant effect on both mortality and hospital readmission rates with reductions of 41 and 32% respectively [10]. A recent Cochrane review confirmed this, albeit to a lesser degree, showing a 13% decrease in all-cause mortality and a 26% decrease in cardiovascular mortality in those attending CR [11].

Despite its proven benefits, CR referral and participation rates still remain low. In a recent Australian study looking at 3212 patients undergoing angiography for AMI, only 48% of patients were referred to CR [12], with numbers in the UK, USA and Europe ranging between 30 and 50% [13]. Additionally, attendance attrition rates in CR programs may reach up to 60% over the course of the program and represents a significant ongoing challenge [14]. Globally there have been cost challenges associated with CR attendance. Suaya JA et al. in the US reported that just over 13% of Medicare patients who had suffered an AMI, and 30% after CABG, received CR services after index hospitalisation [15]. Moreover, there may be a particular subset of the population including women, elderly people, those with significant comorbidities and those with inadequate health insurance coverage that are less likely to participate in or receive CR despite its proven protective effect [15, 16].

CR programs play a vital role in the development of personalized weight optimization strategies and education regarding risks of inactivity and reinforcing the positive benefits of regular physical exercise. Despite guideline recommendations of at least 150 min of moderate-intensity aerobic physical activity or 75 min of vigorous-intensity activity throughout the week, evidence suggests that the majority of individuals do not meet this target [17]. The recent 2014/15 Australian Health survey found that over 70% of the adult population had sedentary or low levels of physical activity, not meeting guideline recommendations [18]. There has been efforts to quantify activity levels. Previously published literature has used pedometer-determined physical activity to identify under-activity and suggest daily targets, with an average step count of less than 5000 steps per day defined as sedentary, 5000–7499 as low activity, 7500–9999 as ‘somewhat active’ and greater than 10,000 steps per day as ‘active’. Individuals who take greater than 12,500 steps per day are classified as ‘highly active’ [19]. Numerous studies have shown the significant benefit of regular physical activity with an up to 30–40% reduction in CV risk when compared to inactive individuals [20, 21].

The new generation of wearable consumer personal activity trackers, incorporating a pedometer and accelerometer, offer unique opportunities to engage and remotely monitor patients engaged in rehabilitation programs with exercise components. These devices, offering sophisticated features such as GPS tracking, heart rate monitoring, activity and sleep tracking with smart phone application integration, give individuals an easy and affordable way to monitor physical activity and energy expenditure with the majority of devices showing acceptable validity of the data recorded [22, 23]. It is estimated that approximately 25 million of these devices were sold in 2015 with worldwide sales expected to increase to 12.6 billion US dollars by 2018 [24]. Wrist-worn monitors are predicted to account for 87% of the devices sold [25].

The use of older generation pedometers is well documented in the medical literature. A systematic review and meta-analysis of 8 randomized controlled trials and 18 observational studies with a total of 2767 patients found an increase in physical activity of 26.9% over baseline as measured by step count in patients using pedometers, with significant reduction in body mass index and blood pressure [26]. An important predictor of increased physical activity was having a daily step goal of 10,000. More recently, a systematic review of 11 studies including 1272 participants across 5 countries concluded that wearing electronic activity monitoring systems lead to an increase in physical activity and decrease in weight, suggesting potentially clinically significant benefits [27]. Indeed, a significant advantage of a mobile device-based study is that participants naturally take the device with them, permitting passive recording of motion however, but its feasibility and validity on a 6-min walk test was questioned in a recent study by McConnell et al. [28]. A combination approach with measures of physical activity through a personal activity tracker and formal serial physical assessments of 6-min walking test perhaps is a more accurate assessment.

One of the limitations to these devices is the documented low rates of long-term engagement for many users [15, 29, 30]. Some have suggested that compliance at two years is approximately 40% [31]. However, despite these concerns, long-term compliance has not been assessed in patients post acute coronary syndrome. It could be argued that given the often life threatening event spurring behaviour change, engagement and compliance may well be higher.

## Aim

The aim of this study is to assess the impact of personal activity trackers on exercise capacity, and improvement of cardiovascular risk factors including blood lipids, blood glucose, mood, weight, waist circumference, body mass index, blood pressure, exercise motivation and quality of life (QOL) in post acute coronary syndrome patients undergoing cardiac rehabilitation,

## Hypotheses

The primary hypothesis is that the use of a personal activity tracker will improve exercise capacity, as measured by an objective 6-min walk test, compared to standard care alone. The secondary hypotheses are that the use of personal activity trackers will result in the improvement in cardiovascular risk factors, namely; improved lipid profile, blood pressure, BMI, weight and waist circumference, HbA1c (where applicable) as well as an improvement in psychological status, mood, exercise motivation and QOL.

## Methods/Design

**Fig. 1:**
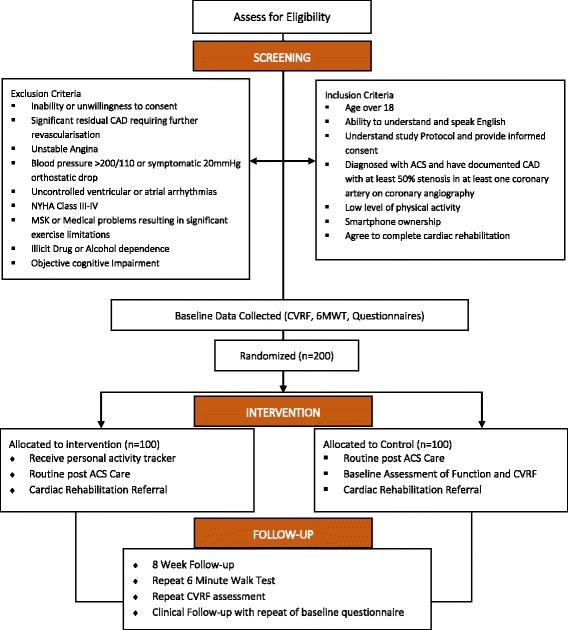
Randomized control trial design and flow

UP-STEP ACS is a prospective single-blinded, two-arm, parallel, randomized control trial with an aim to enrol 200 patients (100 in each arm). The study design is displayed in Fig. 1. This protocol is prepared based on the SPIRIT 2013 statement [32].

### Outcome assessment

#### Primary outcome

The primary outcome is a change in six-minute walk distance that will be measured in a standardized fashion as per the American Thoracic Society Statement 2002 [33]. The initial test will be undertaken during index hospital admission with telemetry monitoring and will be repeated at eight weeks as an outpatient. It will be undertaken by a trained assessor.

#### Secondary outcomes

All secondary outcomes will be measured at baseline and at 8-week follow-up. Table 1 summarizes the secondary outcomes of our study. In addition to those already mentioned, a number of self-reported indices will be recorded at follow-up appointment. Namely, smoking status, date participants returned to work (if applicable), medication adherence, and cardiac rehabilitation attendance and completion, defined as attending all sessions. Any major adverse cardiovascular events (MACE) will be recorded. MACE is defined as the composite of death, non-fatal myocardial infarction, stroke and unplanned revascularization. In addition, cardiac related hospital admissions will also be documented. Both primary and secondary outcomes are outlined in Table 1.

**Table 1:** Primary and Secondary endpoints

Primary Outcome
• Six-minute walk test distance
Secondary Outcome
• Fasting Lipid Levels
• Fasting Glucose
• Resting Blood Pressure
• Resting Heart Rate
• Weight and Body Mass Index (BMI)
• Waist Circumference
• Smoking Status (self-Reported)
• Attendance and adherence to Cardiac Rehabilitation
• Depression/Mood Assessment
• Compliance with Secondary Prevention Pharmacotherapy
• Exercise Motivation (EMI-2)
• Quality of Life

### Eligibility and recruitment

Once potential participants are identified, inclusion and exclusion criteria will be applied. The inclusion criteria are as follows: 1) Patient over the age of 18; 2) Ability to understand and speak English; 3) Ability to understand the study protocol and able to provide informed consent; 4) Admitted to hospital and diagnosed with an acute coronary syndrome and have documented coronary artery disease on coronary angiography (coronary artery stenosis >50% in at least one coronary artery) treated either medically or with percutaneous coronary intervention; 5) Have low level of physical activity, defined by not meeting recommended weekly exercise targets 6) Personal ownership of a smartphone able to run the Fitbit application; 7) Agreement to complete cardiac rehabilitation program.

The exclusion criteria are: 1) Inability or unwillingness to provide informed consent; 2) Significant residual coronary artery disease requiring planned revascularisation; 3) Unstable angina; 4) Blood pressure > 180/110 or symptomatic orthostatic blood pressure decrease >20 mmHg; 5) Uncontrolled ventricular or atrial arrhythmias; 6) Musculoskeletal or medical problems resulting in significant exercise limitation; 7) Significant medical comorbidities with life expectancy of less than one year; 8) New York Heart Association (NYHA) Functional class III-IV; 9) Current illicit drug or alcohol use or dependence that in the opinion of the principal investigator would interfere with adherence; 10) Clinical signs of cognitive impairment. These are outline in Table 2.

**Table 2:** Inclusion and Exclusion criteria

Inclusion Criteria
• Patient over the age of 18
• Ability to understand and speak English
• Ability to understand the study protocol and able to provide informed consent
• Admitted to hospital and diagnosed with an acute coronary syndrome and have documented coronary artery disease on coronary angiography (coronary artery stenosis >50% in at least one coronary artery) treated either medically or with percutaneous coronary intervention
• Have low level of physical activity, defined by not meeting recommended weekly exercise targets.
• Personal ownership of a smartphone able to run the Fitbit application
• Agreement to complete cardiac rehabilitation program.
Exclusion Criteria
• Inability or unwillingness to provide informed consent
• Significant residual coronary artery disease requiring planned revascularisation
• Unstable angina
• Blood pressure > 180/110 or symptomatic orthostatic blood pressure decrease >20 mmHg
• Uncontrolled ventricular or atrial arrhythmias
• Musculoskeletal or medical problems resulting in significant exercise limitation
• Significant medical co-morbidities with life expectancy of less than one year
• New York Heart Association (NYHA) Functional class III-IV
• Current illicit drug or alcohol use or dependence that in the opinion of the principal investigator would interfere with adherence
• Clinical signs of cognitive impairment

### Setting and participants

Recruitment will occur during index hospital admission at Monash Medical Centre and Dandenong Hospital, Melbourne, Australia. Melbourne is the second largest city in Australia with an estimated population of 4.5 million people with healthcare provided predominantly by a public system, with no or minimal charges to patients. Potential participants will be those admitted with an acute coronary syndrome (I.e. Unstable Angina, NSTEMI and STEMI). These patients will be screened and those meeting entry criteria will be invited to participate in the study. Once informed consent is obtained patients will undergo baseline testing prior to randomization as outlined below. Those subsequently allocated to the intervention group will receive their personal activity devices (Fitbit Charge 2) and face-to-face instruction on its proper use prior to hospital discharge.

### Device description

The Fitbit Charge 2 (Fitbit Inc., San Francisco, CA, USA) is a fitness wristband and heart rate monitor with sophisticated photo-plethysmography technology allowing heart rate derived algorithms to estimate energy expenditure based on physical activity intensity, with recent data suggesting acceptable validity [22, 34, 35]. In addition to pedometer and heart rate monitoring, the Fitbit Chargedevice has additional features of automatic activity tracking, connected GPS, automatic reminders to keep active and sleep detection technology. Fitbit Inc. as an in-kind donation will provide the device used in the study. Fitbit Inc. however, will have no involvement in the design, implementation, analysis or interpretation of the results of this study.

### Sample size required

We calculated that 152 participants (76 in both the intervention and control groups) would need to be enrolled for the study to have a statistical power of 80% to detect a clinically important increase of 25 m in the six-minute walk test [21, 22], in the intervention group as compared with the control group, with a two-sided alpha level of 0.05. Accounting for 20% loss to follow-up a minimum of 92 patients in each arm will be required. If the power was to increase to 90% at 5% level of significance 101 patients would be required in each arm of the study. We plan to recruit 100 patients in each arm, totalling 200 patients.

### Ethics and dissemination

This protocol and the informed consent forms were reviewed by the Monash Health Human Research Ethics Committee (HREC) with respect to the scientific content and compliance with applicable research and human subject regulations. Ethics approval was obtained with reference number LNR/17/MonH/62.

Personal health information will be collected from participants and will be stored in a secure databank. During the study a unique number will be used to identify patients with no personal information appearing on any documents or subsequent publications. The information collected will be kept in a secure location and any computer files will be password protected and retained for 15 years. Patients will have the right to access and to request correction of information held as per local state legislation.

### Randomization

Once all screening assessments are completed and eligibility is confirmed patients (*n* = 200) will be randomized in a 1:1 fashion to receiving the personal activity monitor (Fitbit Charge 2) in addition to usual cardiac rehabilitation or the usual cardiac rehabilitation alone through a computer based randomization process (Fig. 1). A computer-generated table will allocate patients in fixed blocks of four. In order to prevent selection bias, the allocation sequence will be concealed from the investigators enrolling and assessing patients utilizing computer randomization software.

Baseline patient demographics and pre-defined screening and assessment, including the 6-min walk test, will occur prior to randomization after patients have given informed consent. Assessors of the primary outcomes will be blinded to the treatment allocation, however participants are not able to be blinded.

### Primary outcome and intervention

This is a mixed-methods behavioural study integrating quantitative and qualitative data to investigate primary and secondary endpoints. The purpose of the intervention is to determine the effect that personal activity trackers have on physical activity as measured by exercise capacity on 6-min walk test post ACS. The personal activity monitor will be provided to participating patients based on their allocation to the intervention group and agreement to participate in cardiac rehabilitation. Patients in the intervention group will download the Fitbit smart phone application, and the devices will be paired in a face-to-face education session. Patients will also receive education on its use, along with assistance in programming exercise targets as per recommended guidelines.

Each participant in the intervention arm will be registered with a cloud storage service via an allocated, de-identified, password protected email address and will be instructed to wear the device during all waking hours. The study will take place over 8 weeks commencing at the time of their discharge from hospital.

### Exercise prescription

Patients in both groups will be encouraged to exercise as per guideline recommendations [17] over the course of the study period. Patients in the intervention arm will be encouraged to meet a target of at least 10,000 steps per day, with the device programmed to this goal. In men who underwent an exercise rehabilitation program, improvement in walking distance (i.e. steps per day) was a strong independent predictor of survival, and a useful guide to prognosis [36] The study coordinators will be able to view these de-identified step counts in real time, however there will be no further interaction with patients in either group until the 8-week follow-up review.

The routine community cardiac rehabilitation program that all will be encouraged to attend encourages patients to engage in regular moderate intensity exercise increasing in a graded degree over a seven-week period centred around a walking program. Moderate intensity is described as exercise that is ‘neither too hard, nor too easy’, with ‘mild shortness of breath, sweating and increased heart rate’ as per the National Australian Heart Foundation recommendations [37].

### Tracking of cardiovascular risk factors

Prior to discharge from hospital patient cardiovascular risk factors will be collected. Blood pressure, cholesterol levels, blood glucose levels, HbA1c, weight, smoking status, waist circumference and BMI will be assessed and recorded along with survey responses as outlined below. Patients will be provided with specific education and given written information as to recommended target levels and strategies to achieve them as per standard of care. There will be no additional blood tests required during the index admission as these parameters represent the standard of care at our institution.

### Screening assessments

Patients will undergo full comprehensive assessment at baseline prior to hospital discharge. This, including all questionnaires, will all be repeated at the 8-week follow-up appointment.

Exercise Capacity – Improvement in exercise capacity (i.e. walking ability) will be assessed via the six-minute walking test (6MWT) in a standardized fashion by trained assessors [33]. Walking distance and walking speed have been demonstrated to be inversely related to cardiovascular events, survival and hospitalisation in patients with cardiovascular disease [38–40].

Physical Activity – The International Physical Activity Questionnaire (Short) [41] will be used to determine physical activity status. Patients will be considered physically inactive if they do not meet the current guideline recommendation for physical activity.

Blood Pressure & Heart Rate – Blood pressure will be measured using an automated sphygmomanometer with pulse oxymetry to measure resting heart rate after a five-minute period of seated rest. Blood pressure will be repeated three times, as per guideline recommendations, with the first measurement discarded and the average taken of the second and third [42].

Blood Tests – Fasting lipids, blood glucose and HbA1c results will be available from inpatient notes. This will be taken as the baseline recording and repeated through the same hospital lab at 8 weeks.

BMI & Other Risk Factors – Body mass index will be derived from the weight (kg) divided by the height (m) squared. Waist circumference, at the level of the umbilicus, will be measured using an anthropometric tape. Smoking status will be recorded, defined as either current, ex-smoker, or lifelong non-smoker with number of pack years recorded where indicated. One pack year will be defined as 20 cigarettes a day, for one year.

Depression - Assessment for depression will be undertaken with the Cardiac Depression Scale – Short Form (CDS-SF) [43].

Exercise Motivation and Quality of Life: QOL assessment will be undertaken via the Short-Form Health Survey Questionnaire (SF-36) [44]. Exercise Motivation will be assessed via the Exercise Motivation Inventory 2 (EMI-2) questionnaire [45].

### Dietary habits

In the intervention group, patients will be encouraged to complete a food diary through the Fitbit smartphone application. This feature allows patients to enter food amounts, time of consumption and gives a running calorie count of both calories consumed and calories expended based on personal anthropometric data entered into the application (I.e. age, height, weight) and activity levels. Patients in the control arm will also be encouraged to keep a similar food diary. Both groups will receive dietary education through cardiac rehabilitation program attendance.

### Cardiovascular health education

All patients will undergo regular cardiac rehabilitation sessions as run by the health service. This incorporates an extensive teaching program around cardiovascular health education, risk factor modification, exercise prescription and dietary advice along with the implications of psychological stress on cardiovascular disease. This program is run in a group setting with family members invited to attend. It is available at various times throughout the week to facilitate high attendance rates. It is run over either a 6-week period incorporating weekly daytime sessions or over 4 weeks incorporating longer evening sessions to facilitate patients with work commitments.

### Cardiac medication list

Patient medications on discharge and at the end of the study will be recorded, with self-reported reasons documented for any alterations.

### Safety considerations

Overall this is a low risk study. Studies have shown patients with pre-existing heart disease have an event rate of one in every 62,000 h of exercise with those without heart disease having a risk of a cardiac event between 1 in 400,000–800,000 h of exercise [46]. Some studies have suggested that strenuous activity is associated with increased risk of cardiac death or complications [47]. However, recent data suggests that near maximal intensity exercise in patients in the week of and thereafter following an uncomplicated acute coronary syndrome is safe [48, 49].

To minimize any risk to patients, those who have a significantly increased risk of suffering harm from this intervention have been excluded from the study. Should any participant experience or report any adverse events or symptoms at any time during the study period they will be advised on how to manage these symptoms and then to seek medical evaluation initially via their general practitioner or emergency department. Patients will be requested to contact the principal investigator about any reported adverse events or symptoms and these will be promptly recorded and managed.

### Standard care

Regardless of treatment assigned all patients will receive the same standard of care post their acute coronary syndromes. This includes specialist cardiology inpatient management, hospital based education, pre-discharge planning, discharge documentation forwarded to their general practitioner, referral to cardiac rehabilitation, a chest pain action plan and cardiologist follow-up organized 4–6 weeks post index event.

### Statistical analysis

The intervention arm will be compared against the control arm on an intention to treat analysis basis. All statistical analyses will be performed using SPSS v23 (IBM, USA). Baseline characteristics will be summarized using descriptive statistics. Paired Student-t test will be used for all normally distributed continuous variables and will be described as mean with standard deviation. Variables not normally distributed will be analysed using the Wilcoxon Signed Rank test. Categorical variables will be described as frequencies and percentages and will be compared using a Pearson chi-squared test.

### Dissemination policy

The results of this study will be made public within 24 months of completion of the trial, with completion being defined as either full enrolment and complete data collection, or collection of enough data to ensure that primary endpoints have sufficient power. We plan to submit a full manuscript to a peer reviewed journal, however the initial release may be an abstract at an appropriate scientific meeting.

## Discussion

To our knowledge the UP-STEP trial will be the first study with the primary aim of determining the impact that personal activity trackers have on exercise capacity in patients following an acute coronary syndrome. The benefits of exercise on general health and in particular patients with coronary artery disease is well documented, however there remain many challenges in translating this into patient adherence. Near maximal exercise testing has been shown to be safe in patients post uncomplicated myocardial infarction in the days prior to discharge, and therefore early introduction of exercise should be strongly encouraged [48–50].

Personal activity trackers have increased in popularity exponentially in recent years and represent a unique opportunity in aiding the management of cardiovascular risk factors. To our knowledge there are no other studies in the literature assessing their efficacy in post ACS patients, and should the results of our study be positive, their use may represent a cost effective and simple intervention for improving outcomes following acute coronary syndrome. We aim to build the scientific evidence regarding their use in the management of secondary prevention in patients with coronary artery disease.

The primary end point of our superiority study is to assess the improvement of exercise capacity as measured by an objective 6-min walk test, with the trial designed to detect a minimal clinically important difference of an increase of 25 m in a standardised 6 min walk test at 8 weeks post hospital discharge [51]. Patient motivation, lifestyle and behaviour changes are crucial elements in the success of cardiac rehabilitation and cardiovascular risk factors modification. The time immediately post ACS for patients is vitally important for recovery, education and introspection, representing a unique opportunity for a positive behavioural change. Numerous studies have shown the favourable effects CR has on behavioural change in this regard [52–54]. We postulate that the use of a personal activity tracker will increase exercise capacity, increase patient exercise motivation and aid in the management of cardiovascular risk factors. Depending on our findings, future work may also investigate the utility of personal activity trackers in the primary prevention of coronary artery disease by reducing rates of physical inactivity, an emerging and previously unrecognised cardiac risk factor.
